# A real-world study on the clinicopathological profile, treatment outcomes and health-related quality of life, anxiety and depression among patients with desmoid tumor at two tertiary care centers in India

**DOI:** 10.3389/fonc.2024.1382856

**Published:** 2024-10-21

**Authors:** Ghazal Tansir, Aparna Sharma, Bivas Biswas, Suryadev Narayan Sah, Somnath Roy, S. V. S. Deo, Sandeep Agarwala, Shah Alam Khan, Sameer Bakhshi, Deepam Pushpam

**Affiliations:** ^1^ Department of Medical Oncology, Institute Rotary Cancer Hospital, All India Institute of Medical Sciences, New Delhi, India; ^2^ Department of Medical Oncology, Tata Medical Center, Kolkata, West Bengal, India; ^3^ Department of Surgical Oncology, Institute Rotary Cancer Hospital, All India Institute of Medical Sciences, New Delhi, India; ^4^ Department of Pediatric Surgery, All India Institute of Medical Sciences, New Delhi, India; ^5^ Department of Orthopedics, All India Institute of Medical Sciences, New Delhi, India

**Keywords:** Desmoid tumor, tyrosine kinase inhibitors, oral metronomic therapy, quality of life, rare diseases

## Abstract

**Background:**

The medical management of DT comprises tyrosine kinase inhibitors (TKIs), hormonal agents, anti-inflammatory drugs with the recently approved gamma secretase inhibitor nirogacestat being the current standard of care. Real-world data on evolving treatment landscapes of DT remains scarce.

**Methods:**

This is a retrospective study of patients with DT registered between 1995 and 2020 at All India Institute of Medical Sciences, New Delhi and Tata Medical Center, Kolkata. Baseline characteristics were analyzed in form of median values and interquartile range. Categorical and continuous variables were compared by chi square and independent samples T- tests respectively. Anxiety, depression and QoL were prospectively measured among 30 patients using Hospital Anxiety and Depression (HADS) and Functional Assessment of Cancer Therapy-General (FACT-G) scales respectively between 2022 to 2023.

**Results:**

200 patients were included with a male-predominant (n=111, 55.5%) population and median age 26.5 (2.5-75) years. Extremity (n=100, 50%) and abdomen (n=65, 32.5%) were commonest primary sites and median of 2 (1–4) lines of treatment were received. First-line included surgery (n=116, 58%), systemic therapy (n=67, 33.5%), radiotherapy (10, n=5%) and active surveillance (n=7, 3.5%). First-line systemic agents included tamoxifen (n=55, 27.5%), imatinib (n=7, 3.5%), sorafenib (n=1, 0.5%) and chemotherapy (n=4, 2%). 2019 onward, 3% and 63% underwent active surveillance and surgery respectively. Best radiological response obtained with tamoxifen was stable disease (SD) (n=76, 59%) and partial response (PR) (n=31, 24.2%). Best radiological response obtained with sorafenib was PR (n=17, 60.7%) and SD (n=9, 32.1%). Thirty patients underwent HADS and FACT-G scale assessment. Mean HADS-Anxiety subscale score was 3.6 (+/-3.9 SD) and HADS-Depression sub-scale score was 2.6 (+/-3.5 SD) with clinically significant anxiety and depression in 2 (6.7%) patients each. The overall mean FACT-G score was 87.5 (+/-12.6 SD) and lower mean physical well-being (p=0.006) and emotional well-being (0.017) scores were significantly associated with higher HADS-anxiety (>/=8) scores.

**Conclusions:**

Assessment of anxiety, depression and QoL are paramount to gauge the psychological impact of DT. This study gives an overview of clinical and management profile of patients with DT in India, with limitations of selection bias, heterogeneous population and small sample size for QoL assessment.

## Background

Desmoid tumor (DT) is a locally aggressive and recurrent connective tissue annual incidence of 5-6 cases per million population (1). This is a rare disease that affects younger patients with a median age of onset at 30-40 years and arises at sites such as extremity, abdomen and abdominal wall (1, 2). The treatment trends of DT have undergone a paradigm change in recent years with chemotherapy, surgical resection and radiotherapy reserved for life or function-threatening scenarios (3). The treatment options for medical management of DT include the gamma secretase inhibitor nirogacestat and tyrosine kinase inhibitors (TKIs) (4, 5). Nirogacestat is the only drug approved by the Food and Drug Administration for the management of DT and is the standard of care (6).

Other therapeutic options are hormonal agents and nonsteroidal anti-inflammatory agents (NSAID) and oral metronomic therapy (OMT). The data for use of OMT while available in refractory solid tumors, is scarce in DT (7–9). The choice of treatment depends on the clinical presentation, location of the tumor, availability of the therapeutic option and adverse event profiles (10–12).

The unique feature of DT is that even though it is a benign disease with low mortality, the morbidity of the disease remains significant (13). Abdominal DT, especially in Familial Adenomatous Polyposis (FAP) produces significant morbidity and mortality with symptoms such as bowel obstruction and ulceration (14). Patients with DT experience significant stress and anxiety owing to the chronic nature of the disease, lack of awareness among medical practitioners regarding and the lack of support groups for DT (15). This necessitates the assessment of their HRQoL as an endpoint in addition to radiological response rates. QoL measures reflect the perspective of patients regarding their symptom burden, impact on their functioning and side effects of treatment. Few studies have explored the HRQoL of patients with DT and have revealed low global health QoL (16).

Data on the evolving treatment landscapes and QoL measures among patients with DT is scarce. Moreover, real-world data on tamoxifen, NSAIDs and OMT are deficient in published literature. We thus performed this study across two tertiary-care centers in India to evaluate the changing trends of treatment, outcomes with various modalities and QoL measured assessment among patients with DT.

## Methods

We conducted a retrospective analysis of consecutive patients with histologically confirmed DT treated at the All India Institute of Medical Sciences (AIIMS), New Delhi and Tata Memorial Center, Kolkata managed between 1995 to 2020. Ethical clearance was obtained from the Institutional Review Boards of respective institutions (IEC-748/02.09.2022, RP-27/2022 and EC/WV/TMC/06/24). Diagnosis of DT made at outside centers was confirmed by the institute pathologist. Patient details including age, sex, Eastern Cooperative Oncology Group (ECOG) performance status (PS), and treatment received were included.

Treatment modalities were classified into surgery, definitive radiotherapy, TKI (including imatinib and sorafenib), tamoxifen (with or without NSAID), chemotherapy (including methotrexate-vinblastine and vincristine doxorubicin cyclophosphamide) and oral metronomic therapy. Oral metronomic therapy comprised of thalidomide 100 milligram (mg) once daily, celecoxib 200 mg twice daily, etoposide 50 mg alternate day to five days/week alternating with cyclophosphamide 50 mg alternate day to 5 days/week for three weeks, six weekly (17). Assessment by Response Evaluation Criteria in Solid Tumors (RECIST) performed, and the radiological responses given by our expert radiologists (18).

A randomly selected subset of the study population aged greater than 18 years at the time of interview was included in the study conducted for assessment of anxiety, depression and QoL at AIIMS. Participants with any cognitive or psychiatric impairment, any communication disability, lack of understanding of English or Hindi language, or unwillingness to provide informed consent were excluded from the study. Institutional review board clearance was obtained prior to commencement of the study. The prospective assessment was conducted between 2022-2023 irrespective of phase of therapy with the help of a study nurse not involved in the treatment of the participant. The interview was conducted in the hospital when the patient reported for their visit. Clinical, epidemiological and treatment details of the participants were noted. FACT-G (Functional Assessment of Cancer Therapy-General) and HADS (Hospital Anxiety and Depression Scale) questionnaires were administered in English or Hindi languages.

The FACT-G is a 27-item questionnaire consisting of the subdomains of physical well being (PWB) with 7 items (score 0-28), social/family well-being (SWB) with 7 items (score 0-28), emotional well-being (EWB) with 7 items and functional well-being (FWB) with 6 items. The answers are given as per Likert scale of 0 (“not at all”) to 4 (“very much”) leading to a possible total score of 0-108. For the responses to be acceptable, more than 80% answers overall and more than 50% in each subdomain had to be given. The scoring of each item was done as per the FACT-G scoring manual (19) and a higher score implied a higher QoL.

The HADS questionnaire is a 14-item questionnaire with 7 questions each targeting anxiety and depression. The answers are to be scored from 0 to 3 and a total score of 11-21 is considered clinically abnormal, 8-10 is borderline and 0-7 is normal (20).

### Statistical analysis

The participants’ baseline clinical characteristics were presented in the form of median (inter-quartile range), mean (standard deviation) and/or frequencies. Categorical variables were compared using the chi-square test and continuous variables following normal distribution were compared using the independent samples T-test. Overall survival (OS) was defined as the duration between date of diagnosis and date of death due to any cause. Kaplan-Meier curve was constructed for depiction of overall survival analyses while Cox proportional hazards model was used to assess the association of clinical variables and survival. Associations between mean QoL and anxiety/depression scores with clinico-radiological factors were analyzed by the Chi-square test. A p-value of <0.05 was considered to be statistically significant. Statistical analysis was carried out using the SPSS software version 26.0.

## Results

### Demographic profile

Two hundred consecutive patients with DT registered between April 1995 and July 2020 were included in the study from both the institutes with baseline characteristics shown in **Table 1**.

**Table 1:** Demographic features of patients with desmoid tumor included in the study.

Demographic feature	Description
Gender: n(%)	Male: 111 (55.5) Female: 89 (44.5)
Median age (95% CI)	26 years (2.5-75)
Age distribution: n (%)	0-25 years: 97 (48.5) 25-50 years: 86 (43) >50 years: 17 (8.5)
ECOG PS: n (%)	0-1: 175 (87.5) 2: 20 (10) 3: 5 (2.5)
Mean tumor dimension (cm) (range)	7 (3-31), SD: 3.40
Sites of disease: n (%)	Head and neck: 8 (4) Thorax: 5 (2.5) Abdomen/trunk: 79 (39.5)^ - Intra-abdominal: 30 (15%), - Abdominal wall/truncal: 49 (24.5%) Extremity: 100 (50) Paraspinal: 4 (2) Others: 4 (2)

^1 patient diagnosed with FAP.

ECOG PS, Eastern Cooperative Oncology Group Performance Status; cm, centimeter; SD, Standard deviation; FAP, Familial Adenomatous Polyposis.

There was male predominance (n=111, 55.5%) and median age was 26 years (range 2.5-75) at the time of diagnosis of DT. Median ECOG PS upon presentation was 1 (range 0-3). The common primary sites of disease consisted of extremity (n=100, 50%), abdomen (n=65, 32.5%), thorax/chest wall (n=5, 2.5%) and with a median symptom duration of 12 months (range 1-140) prior to presentation. Clinical and management details based on anatomic site of disease are provided in **Supplementary Table S6**.

### Treatment modalities with time trends

Patients received a median of two lines (range 1-4) of treatment including surgical resection, systemic therapy, radiotherapy and active surveillance. Systemic therapies (n=220) consisted of tamoxifen (n=127, 57.7%), TKI (n=75, 34%), cytotoxic chemotherapy (n=12, 5.4%) and OMT (17) (n=6, 2.7%) across lines. The first-line treatment (n=200) consisted of surgical resection (n=116, 58%), systemic therapy (n=67, 33.5%), definitive radiotherapy (10, n=5%) and active surveillance (n=7, 3.5%). The types of systemic treatment given in the first-line included tamoxifen (with and without NSAID) (n=55, 27.5%), imatinib (n=7, 3.5%), sorafenib (n=1, 0.5%) and chemotherapy (n=4, 2%). The details of all lines of treatment are provided in **Table 2**.

**Table 2:** Lines of treatment received by the study population.

	Treatment	n (%)
First line (n=200)	Surgery	116 (58)
	Tamoxifen +/- NSAID	55 (27.5)
	Imatinib	7 (3.5)
	Sorafenib	1 (0.5)
	Methotrexate + vinblastine	4 (2)
	Active surveillance	7 (3.5)
	Radiotherapy	10 (5)
Second line (n=119)	Tamoxifen* +/- NSAIDs	58 (48.7)
	Imatinib	26 (21.8)
	Sorafenib	15 (12.6)
	Methotrexate + vinblastine	4 (3.3)
	OMT	2 (1.6)
	VAC	1 (0.8)
	Radiotherapy	12 (10)
	Surgery	1 (0.8)
Third line (n=42)	Tamoxifen** + NSAIDs	12 (28.5)
	Imatinib	13 (30.9)
	Sorafenib	10 (23.8)
	OMT	4 (9.5)
	Methotrexate vinblastine	3 (7.1)
Fourth line	Tamoxifen + NSAIDs	2 (40)
	Sorafenib	2 (40)
	Pazopanib	1 (20)

NSAID, Non-steroidal anti-inflammatory drugs; OMT, Oral metronomic therapy; VAC, Vincristine doxorubicin cyclophosphamide combination.

*3 patients received tamoxifen doses at 60 mg daily.

**3 patients received tamoxifen at 60 mg daily.

**Figure 1:**
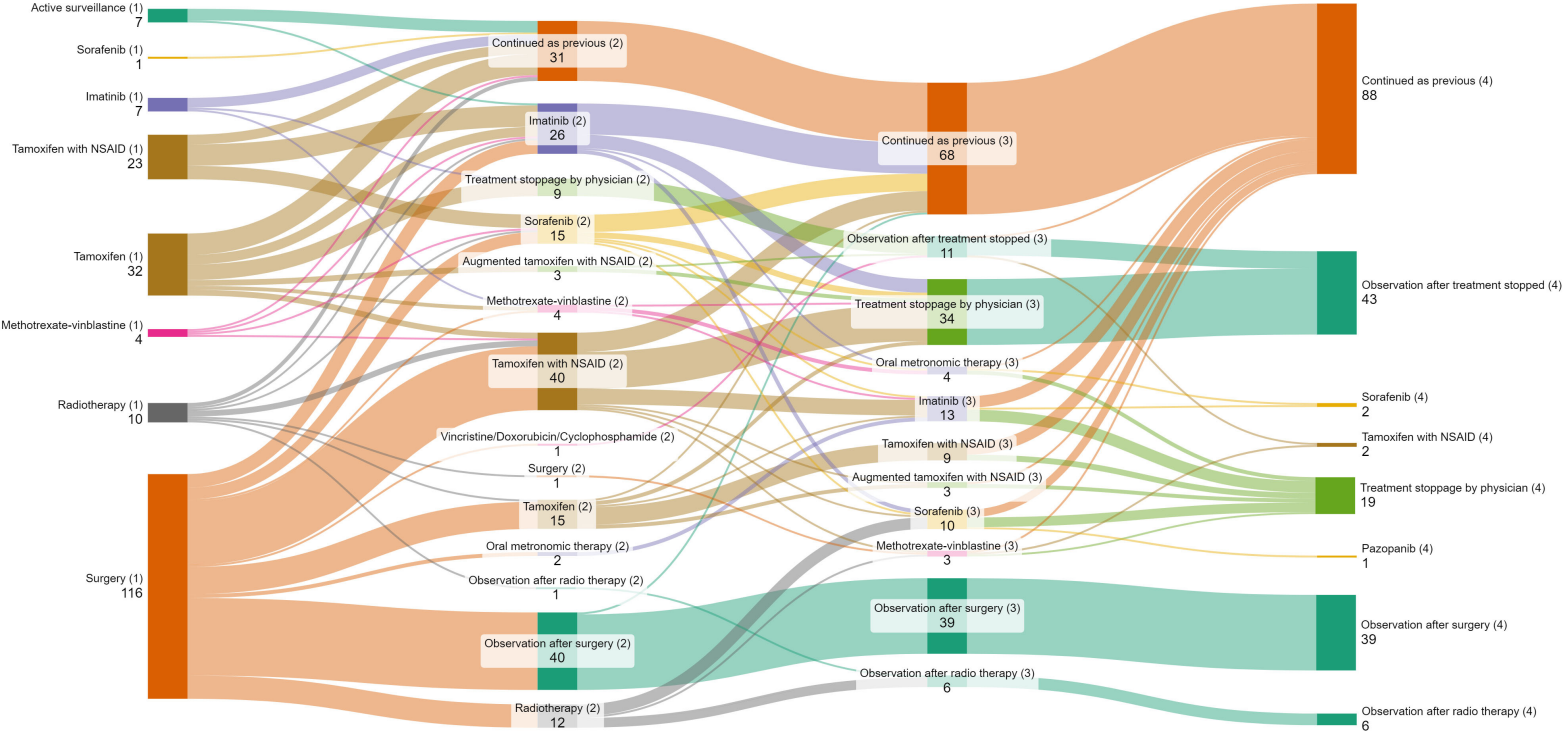
Sankey diagram depicting lines of treatment of patients with desmoid tumor. Bracketed numbers represent each line of therapy (1) First line (2) Second line (3) Third line (4) Fourth line; Non-bracketed numbers represent the number of patients receiving each type of therapy.

**Figure 2:**
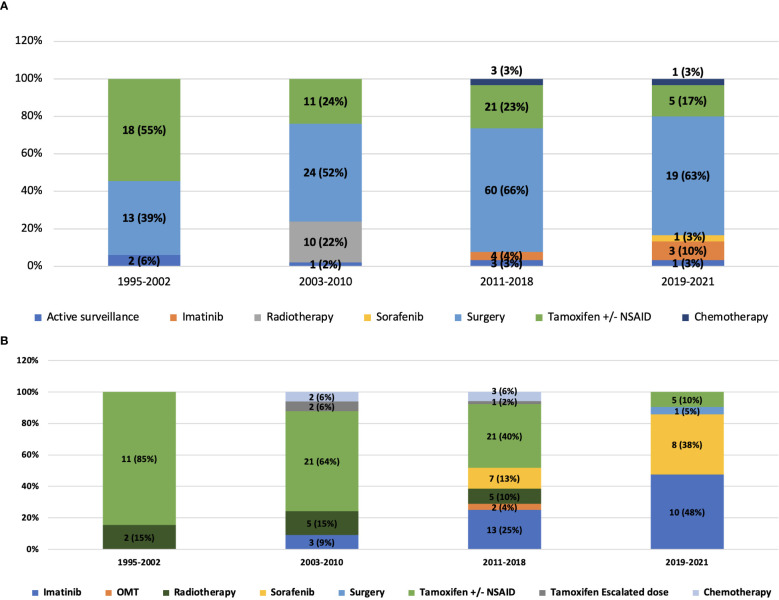
**(A)** Distribution of treatment received in the first line by the study population and its evolution across years of follow-up. **(B)** Distribution of treatment received in the second line by the study population and its evolution across years of follow-up.

In the first line, the proportion of patients undergoing surgical resection across the years was 39% (1995–2002), 52% (2003-2010), 66% (2011-2018) and 63% (2019 onwards). Among patients who progressed after upfront surgery (n=76), 64 (84.3%) underwent medical management and 12 (15.7%) received radiotherapy in the second line. Active surveillance formed a part of first-line therapy in 6% patients in 1995-2002, 2% in 2003-2010, 3% patients each in 2011-2018 and 2019 onwards respectively. 1 (14.2%) patient progressed after active surveillance and was given second-line medical therapy (**Figure 1**). The use of frontline tamoxifen was undertaken in 55% (1995-2002), 24% (2003-2010), 23% (2011-2018) and 17% (2019 onwards) patients. TKIs in the first line setting were used in 4% (2011-2018) and 13% (2019 onwards) patients (**Figure 2A**). In the second line, tamoxifen was used in 85% (1995-2002), 70% (2003-2010), 42% (2011-2018) and 10% (2019 onward) patients. The use of TKI in second line was distributed as 0% (1995-2002), 9% (2003-2010), 38% (2011-2018) and 81% (2019 onwards) (**Figure 2B**).

### Treatment outcomes

The best radiological response according to RECIST v1.1 to systemic treatments in the first line (n=67) was stable disease (38, 80.8%), partial response (n=15, 22.3%) and progressive disease (n=12, 17.9%) (**Table 3**). The best response with systemic therapies in the second line (n=106) was stable disease (n=57, 53.7%), partial response (n=38, 35.8%) and progressive disease (n=11, 10.3%).

**Table 3:** Response rates with each line and type of therapy received by the study population.

	Best response (n, %)
First line medical therapy (N=67)	SD (38, 56.7)
	PR (15, 22.3)
	PD (12, 17.9)
	NE (2, 2.9)
Second line medical therapy (N=106)	SD (57, 53.7)
	PR (38, 35.8)
	PD (11, 10.3)
Third line medical therapy (N=42)	SD (21, 50)
	PR (15, 35.7)
	PD (1, 2.3)
	NE (5, 11.9)
Fourth line medical therapy	SD (2, 40)
(N=5)	PR (3, 60)
Across all lines of treatment:	
Tamoxifen +/- NSAID (N=127)	SD (76, 58.9)
	PR (31, 24.4)
	PD (17, 13.3)
	NE (3, 2.3)
Sorafenib (N=28)	SD (9, 32.1)
	PR (17, 60.7)
	NE (2, 7.1)
Imatinib (N=46)	SD (24, 52.1)
	PR (19, 41.3)
	PD (3, 6.5)
Active surveillance	SD (6, 85.7)
(N=7)	PR (1, 14.3)
Oral metronomic therapy	SD (5,83.3)
(N=6)	NE (1, 16.6)

NSAID, Non-steroidal anti-inflammatory drugs; TKI, Tyrosine kinase inhibitor; SD, Stable disease; PR, Partial response; PD, Progressive disease; NE, Not evaluable; CI, Confidence interval; NR, Not reached; §, chemotherapy, oral metronomic therapy, pazopanib.

Across all treatment lines, the predominant documented best response with tamoxifen was disease stabilization (n=76, 59%) followed by partial response (n=31, 24.2%). Among TKIs across lines, the best response obtained with sorafenib was partial response (n=17, 60.7%) and stable disease (n=9, 32.1%). The best response with imatinib was stable disease (n=23, 51.1%) and partial response (n=19, 41.3%).

Among the 145 patients who were given medical therapy across all line, treatment was discontinued in 61 (42%) patients following a median duration of 73 months (95% confidence interval: 3-300), and followed with observation till the data cut-off on 1st August 2023. No patients who were observed after treatment discontinuation have had disease progression. At a median follow-up of 89 months (95% confidence interval: 70.2-107.7), there were no deaths in our study population.

### Assessment of anxiety and depression

Thirty patients with DT were interviewed using the HADS questionnaire. The interviewees exhibited a female predominance (19, 63.3%) and with a median time of 5 years (range: 1-9 years) from diagnosis. The median age of the patients was 21 years (18–43) at the time of diagnosis of DT. The median tumor dimension was 10 centimeters (4–31) with tumor size of greater than 10 centimeters present in 16 (53.3%) patients.

All the patients responded to more than 90% of the questions of the questionnaires. The mean HADS-Anxiety sub-scale score was 3.6 (+/- 3.9 SD) and HADS-Depression sub-scale score was 2.6 (+/- 3.5 SD) (**Supplementary Table S1**). The mean scores of HADS-depression and anxiety sub-scales were significantly different on univariate analysis among patients according to time from diagnosis (less than versus greater than 5 years) and number of lines of therapy (less than versus greater than 2). Factors not associated with the HADS-Depression scores were gender, age, performance status, tumor dimension (less than 10 centimeters versus greater than 10 centimeters), primary site (extremity and non-extremity), and phase of therapy (observation versus active treatment) (**Supplementary Table S2**). Multivariate analysis did not yield any statistically significant factors associated with HADS-Anxiety and Depression scales (**Supplementary Table S3**). As per the predefined scoring criteria, clinically significant and borderline anxiety was prevalent in 2 (6.7%) and 3 (10%) patients respectively. Clinically significant and borderline depression were present in 2 (6.7%) patients each.

### Quality of life assessment

Thirty patients were interviewed using the FACT-G questionnaire. The median number of unanswered questions in the FACT-G questionnaire was 1 (0-3) and 28 (93.3%) patients responded to more than 90% of the questionnaire. The least answered question (n=19, 63.3% unanswered) in the questionnaire was GS7 of the social well-being subdomain about satisfaction of the participants with their sex life. The mean PWB score was 22.9 (+/- 5.8 SD), SWB score was 20.6 (+/- 2.1 SD), EWB score was 21.2 (+/- 3.9 SD) and FWB was 22.4 (+/- 5.3 SD) with the overall mean FACT-G score of 87.5 (+/- 12.6 SD).

In the Physical well-being sub-scale, the maximum responses of “quite a bit/very much” were given to the questions regarding “I have nausea” (93.3%), “I am forced to spend time in bed” (90%) and “I feel ill” (82.8%). The assessment of the Social/Family well-being yielded that patients obtained “quite a bit/very much” support from family (100%), partner (96.7%) and friends (83.3%). Sexual dissatisfaction was reported in 3 out of 11 respondents (27.3%) answering “not at all/a little bit” sexually satisfied. In the Emotional well-being parameter, patients answered “quite a bit/very much” in terms of nervousness (96.7%), worry about dying (96.7%), sadness (86.7%), losing hope of fighting against their illness (86.7%) and worry about worsening of their condition (83.3%). In the Functional well-being scale, participants answered “quite a bit/very much” to questions on enjoying the things they do for fun (93.3%), being content with their quality of life (93.3%), sleeping well (89.7%), the ability to work (63.3%), and finding their work fulfilling (51.7%).

On univariate analysis, time from diagnosis less than 5 years had significantly positive association with mean FACT-G scores compared to time from diagnosis greater than 5 years (EWB and FWB). Less than 2 lines of therapy had significant positive association compared to greater than 2 lines (PWB and EWB) while observation phase of treatment had significant positive association with mean FACT-G scores compared to active treatment (SWB) (**Supplementary Table S4**). Multivariate analysis did not yield any factors with statistically significant association with FACT-G scores (**Supplementary Table S5**). Lower mean PWB (p=0.006) and EWB (0.017) scores were significantly associated with higher HADS-anxiety (>/=8) score but not significant at the threshold of clinically significant anxiety (>/=11). No association was found between the FACT-G mean subdomain scores with HADS-depression scale.

## Discussion

In this study, we describe the clinical profile and treatment outcomes of patients with DT who were managed at two referral centers of India. This cohort of patients comprised both pediatric and adult patients, with a median age that falls in the age distribution described in previous studies (1). The predominance of extremity and abdominal primary sites in our patients is also in accordance with published literature (2). The gender distribution of our study population, however, was discordant as compared to that published in Western literature (2). While DT is not a malignant condition, this observation could mirror the gender disparities associated with presentation of patients with cancer to healthcare facilities in India (21). We observed that most patients had a good performance status even though they presented to our centers after a symptom duration as high as 12 years. This can be explained by both the chronicity of the disease as well as significant delays in diagnosis due to the rarity of the disease (22). Patients with DT usually experience diagnostic lags due to non-specific symptoms, slow tempo of disease, lack of awareness among physicians as well as pathologic misdiagnoses (23).

Our study covers a population treated over almost 3 decades, allowing an assessment of the evolution of management of DT over the years. Studies have now established that an initial surgical approach does not provide benefit in terms of event-free survival and long-term disease control compared to conservative measures (24). This paradigm shift has now led to surgery having a very limited role in the management of DT as per current guidelines (10). Yet the fact that referrals from surgical disciples remain frequent in our study highlights the need for further iteration that DT is essentially a medically manageable disease. The role of radiotherapy also has been deemed limited, both in the adjuvant and definitive settings (25). The risk of radiation-induced sarcomas in these young patients is an additional factor that restricts the role of radiotherapy to scenarios where other options are exhausted. Active surveillance of asymptomatic patients irrespective of the size and location of tumor is an accepted strategy in current times (26). Due to rarity of disease and lack of awareness among practitioners in peripheral centers of developing countries, patients with DT are initially managed by general surgeons and subjected to surgical resections and then referred to specialized centers. Thus, we find that 60% of our patients had undergone upfront surgery, and that active surveillance was carried out in less than 5% patients only. Though this is a small subset, it is still notable that around 86% patients did not experience disease progression while on surveillance. Thus, active surveillance for DT should be considered as an option for eligible patients after careful assessment by connective tissue tumor experts.

Among systemic therapy options, randomized phase 3 data is available for nirogacestat (5) and sorafenib only (26). While clinical benefit in treatment of DT has been demonstrated by TKIs such as sorafenib and pazopanib, real-world data remains sparse. Variable results have also been observed with the use of low-dose conventional chemotherapy in small studies (27). In the developing world, the paradigm of medical therapies is shifting from anti-hormonal therapies to TKIs with the improved accessibility, affordability and physician awareness of newer drugs such as sorafenib.

This is reflected among our patients also, with the use of tamoxifen/NSAID combination reducing across the years both in first and second lines. Indeed, TKIs were prescribed more frequently after 2010 and comprised more than 80% of second-line systemic treatments. The high rates of disease stabilization with tamoxifen and imatinib among our patients are in concordance with previous studies (28, 29). Sorafenib exhibited overall response rates higher than described in a previous randomized controlled trial (30), but this observation has limited interpretation because only a small number of patients received the drug in our population. While the use of oral chemotherapy has been sparsely documented among DT (7, 31), we used OMT among a few pre-treated patients in this study. With disease stabilization achieved in more than 80% of them, OMT could be explored as a treatment option especially in resource-limited settings. The disease control rate (DCR) obtained with various treatment agents was similar across all groups of medical therapies such as tamoxifen (83.3%), imatinib (93.4%), sorafenib (92.8%) and OMT (83.3%). Hence, the sequencing of therapies could be based on the side effect profile and local availability of the agents.

Assessment of anxiety and depression in our patients yielded a significant association between longer times to diagnosis and number of lines of therapies received. This finding reiterates previous reports of the burden that diagnostic lags and morbidity associated with DT cause (9). The prevalence of clinically significant depression in 6.7% of the interviewed patients compared to another study from our center that found 7.8% patients with DT who fulfilled criteria of major depressive disorder (16). We found a significant impact on the QoL of the patients especially in the aspects of physical, and emotional sub-scales. A majority of patients experienced negative emotions such as nervousness, worry about death and sadness along with physical symptoms of nausea and ill-health. We notably found that anxiety, depression and QoL scores were not significantly different in patients who were on active treatment or observation at the time of interview. Similar findings have also been described in previous analyses; wherein active surveillance did not jeopardize QoL scores among patients with DT compared to those who received active therapy (32). We could also demonstrate that worse emotional and functional scores correlated with greater anxiety, however, the patient numbers are too low to establish significance. The high prevalence of nausea in the patients is an interesting observation in the physical well-being sub-scale. With the caveat of a small sample size, our findings could potentially depict higher degree of adverse events than experienced by the Western population (29). The fear of death was found in close to 97% of the patients, possibly denoting the lower health literacy among Indian patients as demonstrated in previous studies (33). Our findings denote the important concerns in patients with DT in terms of anxiety, depression and QoL deterioration. There is also a need of proper counseling regarding the prognosis and outcomes of this disease to improve their understanding about the disease. The lacunae are expected to be addressed by the recently developed DT-specific GODDESS questionnaire (34).

The limitations of our study include selection bias, single time-point measurement of QoL, anxiety and depression and heterogeneous study population with adult and pediatric patients. Radiological assessment was not carried out at pre-defined intervals and was done on the treating oncologists’ discretion. The small sample size assessed for anxiety, depression and QoL limits the interpretation of the observations, and a larger subset would be desirable for better assessment. Toxicity profile of various treatment agents could not be adequately recorded due to the retrospective nature of the study. However, this is the largest study of its kind to give an overview of the clinical and therapeutic profile of patients with DT. Long follow-up and description of treatment paradigms especially from the developing world are other strengths of our study.

## Conclusions

This study gives a detailed profile of patients with DT managed at two referral centers in India. The evolving treatment patterns with increasing use of TKIs in recent years compared to hormonal agents was demonstrated. There is a limited role of conventional chemotherapy, surgery and radiotherapy in the current era, and active surveillance is an acceptable strategy in selected patients. Though in this real-world study, all types of medical treatments produced similar DCR, the choice of therapy depends on multiple disease and patient-related factors. Systemic treatment options of choice in the current era include sorafenib and nirogacestat, depending on availability. In developing countries, other medical treatments could serve as alternatives as our study demonstrates. The morbidity in patients with DT can be assessed by QoL and scales for anxiety and depression, which can serve as an endpoint for response to treatment. Being a rare disease, a robust referral system should be established to provide patients with DT specialized expertise care for their treatment.

## Data Availability

The original contributions presented in the study are included in the article/**Supplementary Material**. Further inquiries can be directed to the corresponding author.
