# Supplementary material for: A real-world study on the clinicopathological profile, treatment outcomes and health-related quality of life, anxiety and depression among patients with desmoid tumor at two tertiary care centers in India
Source: Front Oncol. 2024 Oct 21;14:1382856. doi: 10.3389/fonc.2024.1382856 (PMC11532177; doi:10.3389/fonc.2024.1382856)
Supplement: Supplementary file 1 [file Table1.docx]

|  | HADS-Anxiety score | HADS-Depression score | FACT-G PWB score | FACT-G SWB score | FACT-G EWB score | FACT-G FWB score |
| --- | --- | --- | --- | --- | --- | --- |
| Mean (+/- SD) | 3.67 (+/- 3.91) | 2.68 (+/- 3.53) | 22.9 (+/- 5.83) | 20.6 (+/- 2.13) | 21.2 (+/- 3.92) | 22.4 (+/- 3.53) |
| Median (range) | 2 (0-13) | 1.58 (0-13) | 25 (9-19) | 20.5 (17.1-26) | 23 (9-24) | 24 (6-22) |

Supplementary Table 1: Assessment of anxiety, depression and quality of life parameters in the study population

Abbreviation: HADS: Hospital Anxiety and Depression scale, FACT-G: Functional Assessment of Cancer Therapy-General, PWB: Physical Well-being, SWB: Social/family well-being, EWB: Emotional well-being, FWB: Functional well-being, SD: Standard deviation.
