# Supplementary material for: A real-world study on the clinicopathological profile, treatment outcomes and health-related quality of life, anxiety and depression among patients with desmoid tumor at two tertiary care centers in India
Source: Front Oncol. 2024 Oct 21;14:1382856. doi: 10.3389/fonc.2024.1382856 (PMC11532177; doi:10.3389/fonc.2024.1382856)
Supplement: Supplementary file 2 [file Table2.docx]

Supplementary Table 2: Univariate and Multivariate analysis of factors associated with anxiety by the HADS questionnaire

|  | Mean HADS-Anxiety score (+/- SD) | 95% confidence interval  (Univariate) | p value  (Univariate) | 95% confidence interval  (Multivariate) | p value  (Multivariate) |
| --- | --- | --- | --- | --- | --- |
| Current age  <30 years (n=16)  >/=30 years (n=14) | 1.66 (1.52)  3.92 (4.06) | -2.57 – 3.91 | 0.91 | -3.61 – 4.868 | 0.75 |
| Gender  Male (n=11)  Female (n=19) | 4.2 (4.70)  3.38 (3.51) | -2.82 – 4.45 | 0.12 | -5.17 – 5.09 | 0.98 |
| Time from diagnosis  <5 years (n=10)  >/=5 years (n=20) | 1.22 (1.30)  4.84 (4.21) | 1.42-5.81 | <0.005 | -0.19 – 1.72 | 0.10 |
| Tumor size  <10 cm (n=14)  >/=10cm (n=16) | 2.53 (3.47)  4.66 (4.11) | -0.82 – 5.07 | 0.15 | -7.01 – 3.30 | 0.44 |
| Primary site  Extremity (n=17)  Non-extremity (n=13) | 3.75 (3.92)  3.58 (4.07) | -3.00 – 3.33 | 0.71 | -3.24 – 5.00 | 0.64 |
| Lines of treatment  <2 (n=13)  >/=2 (n=17) | 1.83 (2.03)  5.06 (4.44) | 0.25-5.00 | 0.001 | -6.44 – 2.14 | 0.29 |
| On observation (n=11)  On therapy (n=19) | 4 (4.52)  3.5 (3.66) | -3.04 – 4.04 | 0.30 | -4.37 – 3.92 | 0.90 |

Abbreviation: SD: Standard Deviation, cm: centimeters, HADS: Hospital Anxiety and Depression Scale
