# Supplementary material for: A real-world study on the clinicopathological profile, treatment outcomes and health-related quality of life, anxiety and depression among patients with desmoid tumor at two tertiary care centers in India
Source: Front Oncol. 2024 Oct 21;14:1382856. doi: 10.3389/fonc.2024.1382856 (PMC11532177; doi:10.3389/fonc.2024.1382856)
Supplement: Supplementary file 3 [file Table3.docx]

|  | Mean HADS-Depression score (+/- SD) | 95% confidence interval  (Univariate) | p value  (Univariate) | 95% confidence interval  (Multivariate) | p value  (Multivariate) |
| --- | --- | --- | --- | --- | --- |
| Current age  <30 years (n=16)  >/=30 years (n=14) | 1.66 (1.52)  3.92 (4.06) | 0.97 – 4.29 | 0.10 | -3.18 – 4.30 | 0.89 |
| Gender  Male (n=11)  Female (n=19) | 4.2 (4.70)  3.38 (3.51) | -2.5 – 4.13 | 0.61 | -5.6 – 4.19 | 0.75 |
| Time from diagnosis  <5 years (n=10)  >/=5 years (n=20) | 1.22 (1.30)  4.84 (4.21) | 1.00 – 4.93 | 0.005 | -0.27 – 1.55 | 0.15 |
| Tumor size  <10 cm (n=14)  >/=10cm (n=16) | 2.53 (3.47)  4.66 (4.11) | -1.5 – 4.02 | 0.35 | -5.4 – 4.42 | 0.82 |
| Primary site  Extremity (n=17)  Non-extremity (n=13) | 3.75 (3.92)  3.58 (4.07) | -2.94 – 2.71 | 0.93 | -3.05 – 4.83 | 0.632 |
| Lines of treatment  <2 (n=13)  >/=2 (n=17) | 1.83 (2.03)  5.06 (4.44) | 0.25 – 5.00 | 0.03 | -6.2 – 2.01 | 0.28 |
| On observation (n=11)  On therapy (n=19) | 4 (4.52)  3.5 (3.66) | -2.76 – 3.48 | 0.94 | -3.99 – 3.94 | 0.98 |

Supplementary Table 3: Univariate and multivariate analysis of factors associated with depression by the HADS questionnaire

Abbreviation: SD: Standard Deviation, cm: centimeters, HADS: Hospital Anxiety and Depression Scale
