# Supplementary material for: A real-world study on the clinicopathological profile, treatment outcomes and health-related quality of life, anxiety and depression among patients with desmoid tumor at two tertiary care centers in India
Source: Front Oncol. 2024 Oct 21;14:1382856. doi: 10.3389/fonc.2024.1382856 (PMC11532177; doi:10.3389/fonc.2024.1382856)
Supplement: Supplementary file 4 [file Table4.docx]

|  | FACT-G PWB score | 95% CI | p value | FACT-G FWB score | 95% CI | p value | FACT-G EWB score | 95% CI | p value | FACT-G SWB score | 95% CI | p value |
| --- | --- | --- | --- | --- | --- | --- | --- | --- | --- | --- | --- | --- |
| Current age  <30 years (n=16)  >/=30 years (n=14) | 27 (5.99)  22.55 (5.99) | -11.65 – 2.75 | 0.66 | 25.33 (3.05)  22.11 (5.52) | -9.93 – 3.49 | 0.33 | 22.66 (2.30)  21.07 (4.06) | -6.08 – 2.90 | 0.38 | 20.5 (0)  20.6 (2.25) | -0.69 – 1.08 | 0.08 |
| Gender  Male (n=11)  Female (n=19) | 22.61 (4.86)  23.21 (6.44) | -5.20 – 4.00 | 0.43 | 20.5 (6.78)  23.5 (4.9) | -7.11 – 1.05 | 0.10 | 21.3 (4.38)  21.15 (3.76) | -2.89 – 3.30 | 0.78 | 20.8 (1.84)  20.5 (2.33) | -1.45 – 1.91 | 0.45 |
| Time from diagnosis  <5 years (n=10)  >/=5 years (n=20) | 24.2  (5.61)  22.3 (5.99) | -6.45 – 2.85 | 0.29 | 25.5 (1.90)  20.9 (5.92) | -7.58 – 1.60 | 0.01 | 22.7 (1.88)  20.5 (4.48) | -5.25 – 0.85 | 0.03 | 21.12 (1.60)  20.45 (2.36) | -2.18 – 0.84 | 0.32 |
| Tumor size  <10 cm (n=14)  >/=10cm (n=16) | 24.5 (6.44)  21.6 (5.09) | -7.13 -1.50 | 0.81 | 23.3 (4.13)  21.6 (6.29) | -5.82 – 2.26 | 0.14 | 21.7 (3.45)  20.8 (2.22) | -3.87 – 2.07 | 0.71 | 20.55 (2.10)  20.78 (2.22) | -1.39 – 1.85 | 0.84 |
| Primary site  Extremity (n=17)  Non-extremity (n=13) | 23.93 (4.88)  21.76 (6.90) | -2.23 – 6.57 | 0.20 | 21.88 (6.44)  23.15 (3.69) | -5.37 – 2.84 | 0.055 | 21.29 (3.27)  21.15 (4.79) | -2.87 – 3.15 | 0.36 | 20.65 (2.28)  20.69 (2.01) | -1.67 – 1.60 | 0.89 |
| Lines of treatment  <2 (n=13)  >/=2 (n=17) | 25.9 (2.88)  20.77 (6.58) | -9.15 –  -1.11 | 0.003 | 24.2 (4.60)  21.02 (5.63) | -7.19 – 0.67 | 0.25 | 22.76 (2.16)  20.05 (4.58) | -5.53 – 0.11 | 0.03 | 20.98 (2.16)  20.43 (2.14) | -2.17 – 1.07 | 0.84 |
| On observation (n=11)  On therapy (n=19) | 22.64 (5.82)  23.2 (5.99) | -5.16 – 4.05 | 0.99 | 21.94 (6.35)  22.72 (.90) | -5.01 – 3.46 | 0.61 | 21.18 (2.31)  21.56 (2.93) | -3.18 – 3.02 | 0.11 | 21.56 (2.93)  20.15 (1.34) | -0.19 – 3.00 | 0.003 |

Univariate analysis of the factors associated with quality of life measures by the FACT-G questionnaire

Abbreviations: CI: Confidence interval, PWB: Physical well-being, FWB: Functional well-being, EWB: Emotional well-being, SWB: Social well-being
