# Supplementary material for: A real-world study on the clinicopathological profile, treatment outcomes and health-related quality of life, anxiety and depression among patients with desmoid tumor at two tertiary care centers in India
Source: Front Oncol. 2024 Oct 21;14:1382856. doi: 10.3389/fonc.2024.1382856 (PMC11532177; doi:10.3389/fonc.2024.1382856)
Supplement: Supplementary file 5 [file Table5.docx]

| Prognostic factor | FACT-G PWB  95% CI | p value | FACT-G FWB 95% CI | p value | FACT-G EWB  95% CI | FACT-G EWB  p value | 95% CI | FACT-G SWB  p value |
| --- | --- | --- | --- | --- | --- | --- | --- | --- |
| Current age  <30 years (n=16)  >/=30 years (n=14) | -6.51 – 4.16 | 0.37 | -7.7 – 3.71 | 0.458 | -1.93 – 6.88 | 0.247 | -3.61 – 1.10 | 0.273 |
| Gender  Male (n=11)  Female (n=19) | -3.1 – 10.26 | 0.26 | -2.53 – 11.38 | 0.193 | -5.95 – 4.72 | 0.80 | -2.54 – 3.16 | 0.819 |
| Time from diagnosis  <5 years (n=10)  >/=5 years (n=20) | -3.45 – 7.55 | 0.96 | -2.03 – 0.56 | 0.245 | -1.43 – 0.55 | 0.35 | -0.75 – 0.30 | 0.378 |
| Tumor size  <10 cm (n=14)  >/=10cm (n=16) | -3.77 – 10.98 | 0.84 | -7.95 – 6.05 | 0.775 | -5.00 – 5.73 | 0.88 | -3.41 – 2.32 | 0.689 |
| Primary site  Extremity (n=17)  Non-extremity (n=13) | -4.23 – 9.57 | 0.45 | -4.76 – 6.42 | 0.755 | -5.03 – 3.54 | 0.71 | -2.65 – 1.93 | 0.739 |
| Lines of treatment  <2 (n=13)  >/=2 (n=17) | -9.15 –  -1.11 | 0.003 | -7.19 – 0.67 | 0.25 | -5.53 – 0.11 | 0.03 | -2.17 – 1.07 | 0.84 |
| On observation (n=11)  On therapy (n=19) | -5.16 – 4.05 | 0.99 | -5.01 – 3.46 | 0.61 | -3.18 – 3.02 | 0.11 | -0.19 – 3.00 | 0.003 |

Supplementary Table 5: Multivariate analysis of the factors associated with quality of life

measures by the FACT-G questionnaire

Abbreviations: CI: Confidence interval, PWB: Physical well-being, FWB: Functional well-being, EWB: Emotional well-being, SWB: Social well-being
