# Supplementary material for: A real-world study on the clinicopathological profile, treatment outcomes and health-related quality of life, anxiety and depression among patients with desmoid tumor at two tertiary care centers in India
Source: Front Oncol. 2024 Oct 21;14:1382856. doi: 10.3389/fonc.2024.1382856 (PMC11532177; doi:10.3389/fonc.2024.1382856)
Supplement: Supplementary file 6 [file Table6.docx]

| Primary site (n, %) | Gender distribution  (n, %) | Median age (years) (range) | Median symptom duration (months) (range) | Median tumor size (centimetres) (range) | First-line treatment (n, %) | Second-line (n, %) | Third-line (n, %) | Fourth-line (n, %) |
| --- | --- | --- | --- | --- | --- | --- | --- | --- |
| Extremity (n=100, 50%) | Male (60, 60%) Female (40, 40%) | 21  (2.5-62) | 12  (1-140) | 7  (4-31) | Surgery (54, 54%)  Tamoxifen +/- NSAID (34, 34%)  Radiotherapy (7, 7%)  Methotrexate-vinblastine (2, 2%)  Active surveillance (2, 2%)  Imatinib (1, 1%) | Imatinib (11, 11%)  Tamoxifen +/- NSAID (38, 38%)  Sorafenib (8, 8%)  Radiotherapy (4, 4%)  Surgery (1, 1%)  VAC (1, 1%)  Methotrexate-vinblastine (1, 1%) | Tamoxifen + NSAID (10, 10%)  Imatinib (7, 7%)  Sorafenib (4, 4%)  Oral metronome therapy (2, 2%)  Methotrexate-vinblastine (2, 2%) | Tamoxifen + NSAID (2, 2%)  Pazopanib (1, 1%)  Sorafenib (1, 1%) |
| Intra-abdominal (n=30, 15%) | Male (12, 40%)  Female (18, 60%) | 31  (3-75) | 10  (1-72) | 7  (4-30) | Surgery (19, 63.3%)  Tamoxifen +/- NSAID (6, 20%)  Imatinib (3, 10%)  Sorafenib (1, 3.3%)  Radiotherapy (1, 3.3%) | Imatinib (7, 23.3%)  Radiotherapy (4, 13.3%)  Sorafenib (3, 10%)  Methotrexate-vinblastine (2, 6.6%)  Oral metronomic therapy (1, 3.3%) | Sorafenib (3, 10%)  Imatinib (2, 6.6%) | NA |
| Abdominal wall  (n=15, 7.5%) | Male (18, 60%)  Female (12, 40%) | 29  (22-63) | 11  (1-40) | 7  (4-9) | Surgery (9, 60%)  Active surveillance (2, 13.3%)  Tamoxifen +/- NSAID (2, 13.3%)  Imatinib (1, 6.6%)  Methotrexate-vinblastine (1, 6.6%) | Imatinib (2, 13.3%)  Radiotherapy (1, 6.6%)  Oral metronomic therapy (1, 6.6%)  Tamoxifen + NSAID (1, 6.6%) | Imatinib (2, 13.3%)  Sorafenib (1, 6.6%) | Sorafenib (1, 6.6%) |
| Trunk (n=34, 17%) | Male (19, 56%) Female (15, 44%) | 28  (5-63) | 12  (1-48) | 6 (4-15) | Surgery (19, 55.8%)  Tamoxifen + NSAID (7, 20.5%)  Active surveillance (3, 8.8%)  Imatinib (2, 5.9%)  Radiotherapy (2, 5.9%)  Methotrexate-vinblastine (1, 2.9%) | Tamoxifen +/- NSAID (12, 35.2%)  Imatinib (3, 8.8%)  Radiotherapy (3, 8.8%)  Sorafenib (3, 8.8%)  Methotrexate-vinblastine (2, 5.8%) | Tamoxifen + NSAID (2, 5.9%)  Sorafenib (2, 5.9%)  Oral metronome therapy (2, 5.9%)  Methotrexate-vinblastine (1, 2.9%)  Imatinib (1, 2.9%) | NA |
| Head and neck (n=8, 4%) | Male (3, 38%)  Female (5, 62%) | 23  (5-53) | 5  (3-36) | 4.5 (3-11) | Surgery (7, 87.5%)  Tamoxifen (1, 12.5%) | Tamoxifen + NSAID (3, 38%)  Imatinib (1, 12.5%)  Sorafenib (1, 12.5%) | NA | NA |
| Thorax (n=5, 2.5%) | Male (2, 40%)  Female (3, 60%) | 38  (28-63) | 6  (2-12) | 5 (4-10) | Surgery (3, 60%)  Tamoxifen (2, 40%) | Tamoxifen + NSAID (1, 20%) | NA | NA |
| Paraspinal (n=4, 2%) | Male (4, 100%) Female (0, 0%) | 31  (22-43) | 12  (10-72) | 7 (5-7) | Tamoxifen (3, 75%) Surgery (1, 25%) | Tamoxifen (2, 50%) Imatinib (1, 25%) | Imatinib (1, 25%) | NA |
| Others (n=4, 2%) | Male (2, 50%) Female (2, 50%) | 24  (13-47) | 10  (8-18) | 7 (6-11) | Surgery (4, 100%) | Imatinib (1, 25%) | NA | NA |

**Supplementary Table 6**: Description of clinical and treatment details of patients with desmoid tumor according to anatomic site of disease

Abbreviations: NSAID: Nonsteroidal anti-inflammatory drugs, NA: Not applicable
